# Can Non-invasive Ventilation Modulate Cerebral, Respiratory, and Peripheral Muscle Oxygenation During High-Intensity Exercise in Patients With COPD-HF?

**DOI:** 10.3389/fcvm.2021.772650

**Published:** 2022-01-31

**Authors:** Cássia da Luz Goulart, Flávia Rossi Caruso, Adriana Sanches Garcia de Araújo, Sílvia Cristina Garcia de Moura, Aparecida Maria Catai, Piergiuseppe Agostoni, Renata Gonçalves Mendes, Ross Arena, Audrey Borghi-Silva

**Affiliations:** ^1^Cardiopulmonary Physiotherapy Laboratory, Physiotherapy Department, Federal University of Sao Carlos, UFSCar, Sao Carlos, Brazil; ^2^Cardiovascular Physical Therapy Laboratory, Physiotherapy Department, Federal University of São Carlos, Sao Carlos, Brazil; ^3^Cardiovascular Section, Department of Clinical Sciences and Community Health, Centro Cardiologico Monzino, University of Milano, Milan, Italy; ^4^Department of Physical Therapy, College of Applied Health Sciences, University of Illinois at Chicago, Chicago, IL, United States

**Keywords:** blood flow muscle, heart failure, COPD, cardiovascular physiology, oxygen consumption

## Abstract

**Aim:**

To evaluate the effect of non-invasive positive pressure ventilation (NIPPV) on (1) metabolic, ventilatory, and hemodynamic responses; and (2) cerebral (Cox), respiratory, and peripheral oxygenation when compared with SHAM ventilation during the high-intensity exercise in patients with coexisting chronic obstructive pulmonary disease (COPD) and heart failure (HF).

**Methods and Results:**

On separate days, patients performed incremental cardiopulmonary exercise testing and two constant-work rate tests receiving NIPPV or controlled ventilation (SHAM) (the bilevel mode—Astral 150) in random order until the limit of tolerance (Tlim). During exercise, oxyhemoglobin (OxyHb+Mb) and deoxyhemoglobin (DeoxyHb+Mb) were assessed using near-infrared spectroscopy (Oxymon, Artinis Medical Systems, Einsteinweg, The Netherlands). NIPPV associated with high-intensity exercise caused a significant increase in exercise tolerance, peak oxygen consumption ($\overset{\cdot }{V}{\text{O}}_{2}$ in mlO_2_·kg^−1^·min^−1^), minute ventilation peak (${\overset{\cdot }{V}}_{\text{E}}$ in ml/min), peak peripheral oxygen saturation (SpO_2_, %), and lactate/tlim (mmol/s) when compared with SHAM ventilation. In cerebral, respiratory, and peripheral muscles, NIPPV resulted in a lower drop in OxyHb+Mb (*p* < 0.05) and an improved deoxygenation response DeoxyHb+Mb (*p* < 0.05) from the half of the test (60% of Tlim) when compared with SHAM ventilation.

**Conclusion:**

Non-invasive positive pressure ventilation during constant work-rate exercise led to providing the respiratory muscle unloading with greater oxygen supply to the peripheral muscles, reducing muscle fatigue, and sustaining longer exercise time in patients with COPD-HF.

## Introduction

The coexistence of chronic obstructive pulmonary disease (COPD) and heart failure (HF) typically results in severe impairments in the cardiorespiratory fitness (CRF), including exercise capacity, ventilatory efficiency, oxygen supply to working muscle, and peripheral muscle function. Due to the multisystem pathophysiology brought about by COPD and HF combined, abnormalities observed during exercise testing are often greater compared with patients presenting with either chronic condition in isolation. While cardiac and pulmonary functions clearly and significantly contribute to the impaired CRF in these patients, skeletal muscle abnormalities brought about by the COPD-HF disease processes cannot be overlooked (1–4).

Exercise intolerance may be directly associated with the increased ventilatory response to metabolic demand (5, 6). Goulart et al. (7) found that the patients with COPD-HF have a marked exercise intolerance with greater deoxygenation of both the respiratory and peripheral musculature when compared with HF patients. Therefore, interventions aimed at improving the peripheral muscle dysfunction, unloading respiratory muscles during exercise, and local energy supply to the locomotor muscles are of great importance. In this context, the employment of non-invasive positive pressure ventilation (NIPPV) may prove useful in improving exercise tolerance (8) by increasing blood flow and oxygenation to muscle groups integral to the exercise response (4). A major pathophysiological consequence in resting cerebral oxygenation is found in patients with coexistence of COPD-HF and these abnormalities are accentuated during exercise (9).

Borghi-Silva et al. (4) observed that the respiratory muscle unloading reduced respiratory muscle blood flow requirements with a consequent redistribution of cardiac output to the exercising peripheral muscle when proportional assisted ventilation was applied to patients diagnosed with COPD or HF (10). However, to our knowledge, no studies have focused on the effect of bilevel NIPPV on metabolic and ventilatory responses during high-intensity exercise in patients with coexisting COPD and HF.

Hypothesizing the positive effects of NIPPV on the exercise response in a multimorbid patient population with often severely compromised CRF (i.e., COPD-HF) served as the basis for the current study. Specifically, the aim of this study was to evaluate the effect of NIPPV on (1) metabolic, ventilatory, and hemodynamic responses; and (2) cerebral (Cox), respiratory, and peripheral oxygenation when compared with SHAM ventilation in patients with coexisting COPD and HF during high-intensity exercise. We hypothesized that NIPPV is able to improve the oxygen consumption and ventilation during exercise, in addition to causing an unload of the respiratory muscles, providing greater oxygen availability for peripheral muscles; additionally, we believe that NIPPV will provide less cerebral oxygen extraction during exercise.

## Methods

### Design

This is a cross-over, double blind, controlled study. This study adhered to the Consolidated Standards for Reporting (*CONSORT*) (11), was approved by the local ethics committees (Protocol Number: 91088318.7.1001.5504) of the University of Sao Carlos, São Paulo, Brazil. All patients signed a written informed consent statement prior to participation.

### Subjects

All patients included in this study were 50 years or older with a confirmed coexisting diagnosis of COPD and HF. A clinical diagnosis of COPD was confirmed by the pulmonary function testing [forced expiratory volume in 1 s (FEV_1_)/forced vital capacity (FVC) ratio of <0.7; FEV_1_ < 60% of predicted] and, HF with reduced left ventricular ejection fraction (LVEF) was confirmed by a cardiologist considering LVEF < 50% by ECG and *New York Heart Association Functional Classification* (*NYHA*) I–IV.

Exclusion criteria consisted of (1) musculoskeletal disorders or neurological conditions affecting the ability to exercise; (2) deterioration of clinical status requiring hospitalization 3 months before the study; (3) diagnosis of malignant disease; (4) implantable pacemaker; (5) myocardial infarction (3 months before the study); and (6) complex cardiac arrhythmias.

### Experimental Procedures

The experimental procedure of the current study has been published previously (8). All patients underwent a pulmonary function exam analyzed by a pulmonologist, an ECG analyzed by a cardiologist, and followed by a clinical assessment. Every patient completed the comprehensive evaluation process in 4 days as follows: (1) visit 1: clinical evaluation by a physician and physical therapist, followed by a lung function test and Doppler echocardiography; (2) visit 2: cardiopulmonary exercise testing (CPET); (3) visit 3: familiarization of NIPPV and SHAM with a minimal interval of 48 h between them; and (4) visit 4: two high-intensity constant load exercise sessions (CLE) (SHAM and NIPPV) applied on the same day with an interval of 1 h between them. The interval between the incremental CPET and CLE was 7 days. The intensity for the high-intensity CLE sessions was set at 80% of the peak CPET work rate (WR).

Patients were randomized into two groups with a 1:1 block allocation for the NIPPV or SHAM group. The order of which was determined by randomization, using an envelope, by a researcher not involved in the study. The examiners responsible for evaluations as well as the patients were blinded to the intervention.

### Measurements

#### Pulmonary Function

Spirometry, static lung volumes, and pulmonary resistance were performed using the complete pulmonary function test (MasterScreen™ Body Plethysmograph, Germany), pre- and post-bronchodilator. The FEV_1_ (L), FVC (L), FEV_1_/FVC (L), total lung capacity (TLC, L and %), residual volume (RV, L and %), RV/TLC (%), and inspiratory capacity (IC, L, and %) were obtained (12). Percentage-predicted values were determined in accordance with the recommendations proposed by Alberto et al. (13). The severity of COPD was based on the *Global Initiative for Chronic Obstructive Pulmonary Disease* (GOLD) classification (12).

#### Doppler Echocardiography

Patients underwent a 2D echocardiogram using an iE33 system (Philips, Andover, MA, USA) with a 2–5 MHz matrix transducer and tissue Doppler imaging software. Quantification of the cardiac chambers was performed according to the *American Society of Echocardiography* (14). For this study, LVEF was a primary measure of interest.

#### Cardiopulmonary Exercise Testing

The CPET was performed on a cycle ergometer with electromagnetic braking (CORIVAL V3, Lode BV, Groningen, The Netherlands), and the respiratory gas analysis was measured breath-by-breath using a computer-based system (ULTIMA, MedGraphics–Breeze, St. Paul, MN, USA). The CPET protocol followed the following stages: (1) 5 min of rest; (2) 1 min warm-up at free-wheel with 60 rpm; (3) incremental phase (5–10 W/min, ramp protocol); (4) 1 min active cool-down at free-wheel; and (5) 5-min passive cool-down in the sitting position (15). The test was terminated when patients pedaled at their maximum possible effort level (physical exhaustion) or presented with the established termination criteria, such as angina, electrocardiographic evidence of ischemia, or malignant arrhythmias (ventricular tachyarrhythmia, bigeminy, and arise bundle branch block) (16).

#### Constant Workload Exercise

Then, 7 days after the incremental CPET, patients performed high-intensity exercise during two CLE (SHAM and NIPPV) sessions on a cycle ergometer with electromagnetic braking (CORIVAL V3, Lode BV, Groningen, The Netherlands) with respiratory gas analysis measured breath-by-breath by using a computer-based system (ULTIMA, MedGraphics–Breeze, St. Paul, MN, USA). After baseline assessment at rest, the load was set at 80% of peak CPET work rate (WR) until Tlim, which was turned on 20 min before the test and then calibrated before each test. Pulmonary gas exchange and ventilatory variables were obtained from calibrated signals derived from gas analyzers and a pneumotachograph. The protocol consisted of the following stages: (1) 5-min rest period while sitting on the cycle ergometer; (2) constant phase at 60 rpm; and (3) 5-min passive cool-down in the sitting position. Tlim was defined as the time point at which the patients signaled to stop exercising or could not maintain the required pedaling rate for 10 s, despite being encouraged by the investigators (17). The test was terminated when patients were pedaling at their maximum possible effort level or presented with established termination criteria, such as angina or electrocardiographic evidence of ischemia (16). NIPPV and SHAM were applied in the final 2 min of rest and during exercise.

To avoid interference during the analysis, ULTIMA for ventilatory, metabolic, cardiac, and hemodynamic measurements, Physioflow for cardiac output, and near-infrared spectroscopy (NIRS) to assess the cerebral, respiratory, and peripheral oxyhemoglobin (OxyHb+Mb) and deoxyhemoglobin (DeoxyHb+Mb) were activated at the same time at the beginning of the CLE.

#### Ventilatory, Metabolic, Cardiac, and Hemodynamic Measurements

During CLE, the following parameters were measured: (1) peak systolic and diastolic blood pressure (SBP and DBP) (mmHg); (2) peak oxygen consumption ($\overset{\cdot }{V}{\text{O}}_{2}$) (in ml/min and mlO_2_ kg^−1^ min^−1^); (3) carbon dioxide production $\overset{\cdot }{V}$CO_2_ (ml/min); (4) minute ventilation peak (${\overset{\cdot }{\text{V}}}_{\text{E}}$) (ml/min); 5) WR (W); (6) HR at rest and peak (bpm); (7) peak peripheral oxygen saturation (SpO_2_, %); and (8) Borg dyspnea and leg effort 0–10. The $\overset{\cdot }{V}{\text{O}}_{2}$ (in ml/min and mlO_2_ kg^−1^ min^−1^), $\overset{\cdot }{V}$CO_2_ (ml/min), and ${\overset{\cdot }{V}}_{E}$ (ml/min) were reported as 20-s averaged data. Capillary samples were collected from the fingertip for blood lactate measurements (mEq/L) at rest and exercise cessation (Yellow Springs 2.700 STAT Plus, Yellow Springs Instruments, OH, USA).

Cardiac output (L/min) was measured non-invasively throughout the tests using an impedance cardiography device (PhysioFlow PF-05, Manatec Biomedical, France). Five electrodes were placed, three of which are the current electrodes and two that detect voltage changes; the detected voltage is proportional to the tissue impedance. This technique has high reproducibility and accuracy when compared with invasive hemodynamic monitoring techniques (18). We tested the accuracy of CO analysis in a previous study and observed that the system was sensitive to detect small changes with acceptable accuracy (within <10% for all readings) (18). We analyzed the 12 most stable points at the end of the exercise.

#### Non-invasive Positive Pressure Ventilation

Non-invasive positive pressure ventilation was applied through two pressure levels (bilevel), by a well-adjusted facial mask (Astral 150, Resmed, Brazil), with pressure defined individually in a preliminary visit for each patient. The pressures were titrated within the range of 8–12 cmH_2_O for inspiratory pressure (IP) and 4–6 cmH_2_O for positive end-expiratory pressure (PEEP). Bilevel provides ventilatory assistance of inspiratory (IPAP) and expiratory pressures (EPAP) to reduce the work of breathing (19).

SHAM ventilation was applied with a minimum IPAP (5 cmH_2_O) and EPAP (2 cmH_2_O). Before performing CLE, one of the examiners randomized, in an opaque and sealed envelope, the order of the tests (SHAM and NIPPV). This same examiner was a trained physiotherapist, who was not involved in the study and selected the ventilatory strategy to be applied initially. The other two evaluators and the patients were unaware of the ventilation mode to be applied (8). Before data collection, the system was calibrated while taking body mass, blood pressure, and age values. The verification of the correct signal quality was performed by visualizing the ECG tracing and its first derivative and the impedance waveform (DZ) with its first derivative (dZ/dt).

**Figure 1:**
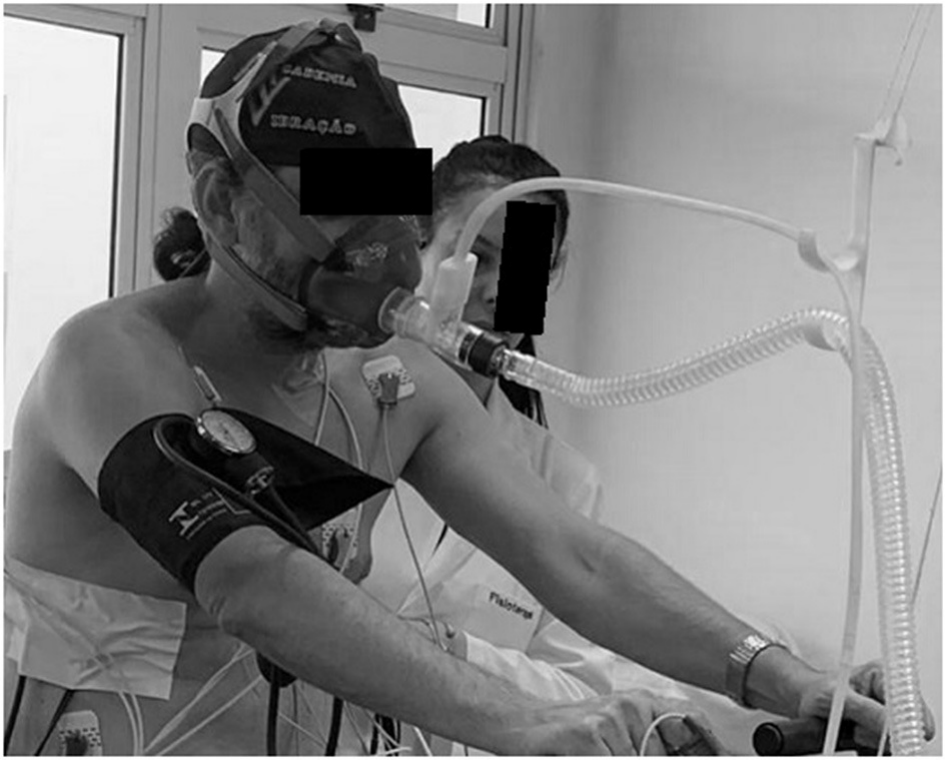
Protocol of our ventilation study adapted with the gas analysis measured breath-by-breath in which the trachea was connected with the pneumotach.

Figure 1 illustrates how the protocol of our ventilation study was adapted with the gas analysis measured breath-by-breath in which the trachea was connected with the pneumotach.

#### Near-Infrared Spectroscopy

During CLE, NIRS (Oxymon, Artinis Medical Systems, Einsteinweg, The Netherlands) data sampled at 250 Hz *via* an online data acquisition system Oxysoft (Artinis Medical Systems, The Netherlands) was performed. Transmitter light and receiver fiber were separated by 40 mm, corresponding to ~20 mm of penetration depth. This method allows dynamic evaluation of relative blood concentration changes of oxyhemoglobin (OxyHb+Mb) and deoxyhemoglobin (DeoxyHb+Mb) (7).

The probes were secured with adhesive tape and placed on (1) the lateral side of the thigh—vastus lateralis (12–14 cm above the proximal border of the patella and 5 cm lateral to the midline of the thigh); (2) the external intercostal muscle (over left 7th intercostal space, anterior axillary line); and (3) the prefrontal region, ~3 cm from the midline of the forehead, above the supraorbital. Before each test, the muscular NIRS probes were calibrated according to manufacturer specifications. The deltas of the variables analyzed by NIRS at the cerebral, peripheral, and respiratory muscles were calculated during CLE, in seconds, divided into deltas (20, 40, 60, 80, and 100% of Tlim), subtracted from the value obtained at rest (60 s of the most stable data). This technique has been validated during the exercise in adults, due to the permeability of near infrared light in biological tissue (20).

We use the differential pathlength factor (DPF) filter since the DPF correction forms the average distance of light propagation detected in the vastus lateralis, the external intercostal muscle, and the prefrontal region (20). The lasers in the NIRS program were activated in sequence for 20 at 100 ms intervals, while the signal processing produces values for attenuation (corrected for any backlight) (20).

### Statistical Analysis

The required number of patients to be assessed (*n* = 14, crossover study) was calculated considering the Tlim of CLE as the main outcome (10), assuming a risk of α of 5% and β of 20% with an expected improvement of 74 s between NIPPV vs. Sham. Shapiro–Wilk test was used to verify the normality of data. Descriptive data is listed as a mean, SD, and percentage (%).

To test the hypothesis of our study, we applied (1) the Student's *t*-test for comparison of metabolic, ventilatory, and cardiovascular/hemodynamics variables between NIPPV and SHAM; and (2) the two-way analysis with multiple comparisons, according to ventilation (NIPPV *vs*. SHAM), OxyHb+Mb and DeoxyHb+Mb effect (20, 40, 60, 80, and 100%) for cerebral, respiratory, and peripheral muscle oxygenation. All tests were made in GraphPad Prism 7.0 (GraphPad Software, CA, USA) with statistical significance set at *p* ≤ 0.05.

## Results

**Figure 2:**
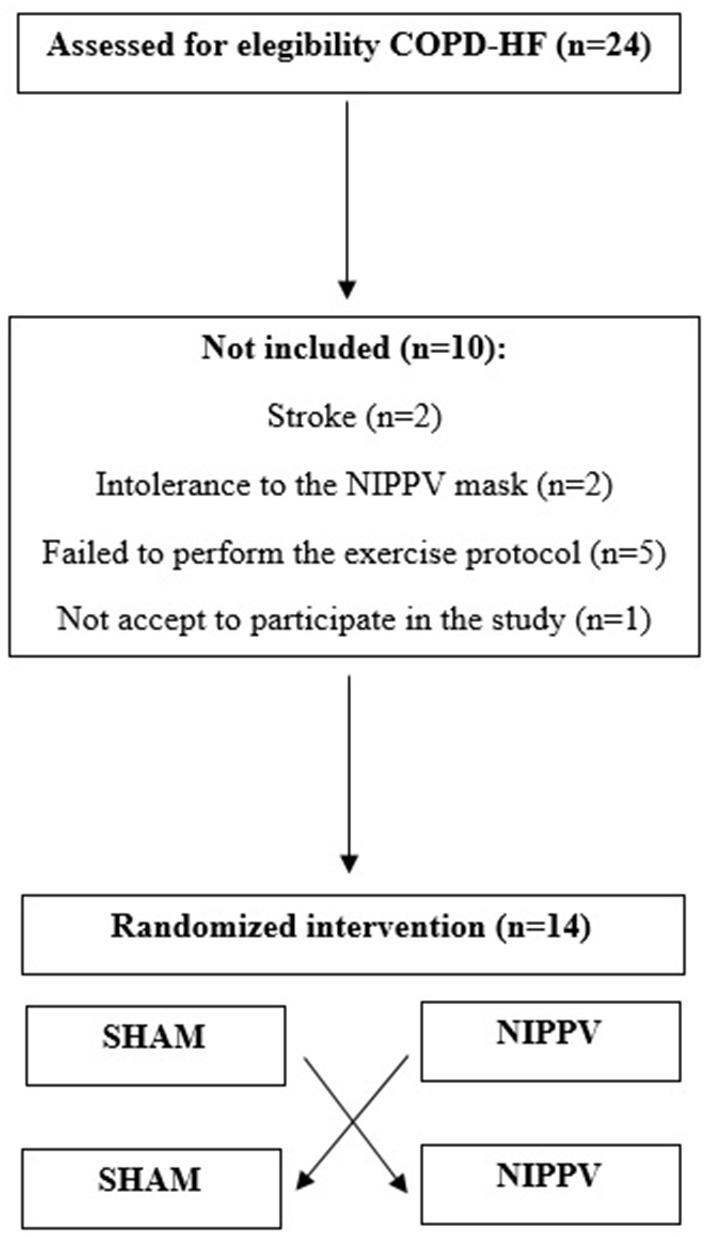
Flow diagram representing the sample recruitment and loss.

In this study, 24 patients were initially recruited and 10 were excluded. Therefore, 14 patients diagnosed with COPD-HF completed all visits and protocols proposed for the current study (Figure 2).

### General Characteristics

In the current study, 14 male patients were included, age 70 ± 7 years, LVEF 41 ± 9%, nine had an LVEF between 40 and 50% with the remaining 5 below 40%, expected reductions in FEV_1_/FVC (L) and IC (%), as well as increases in VR (%) and RV/TLC (%) due to COPD. In addition, COPD severity was classified as mild (7, 50%), moderate (5, 35%), and severe (2, 14%), and NYHA I (5, 35%), II (6, 42%), and III (3, 22%) demonstrating a mild-moderate multimorbidity phenotype cohort. The medications used were beta-blockers 14 (100%), beta-agonists 14 (100%), diuretics 11 (79%), and inhaled corticosteroid 8 (54%).

### Effect of NIPPV and SHAM as an Adjunct of High-Intensity Exercise on Metabolic, Ventilatory, and Cardiovascular/Hemodynamics

Non-invasive positive pressure ventilation associated with high-intensity exercise caused a significant increase in exercise tolerance, $\overset{\cdot }{V}{\text{O}}_{2}$ (ml/min) peak, $\overset{\cdot }{V}{\text{O}}_{2}$ (mlO_2_ kg^−1^ min^−1^) peak, *V°C*O_2_ (ml/min) peak, and $\overset{\cdot }{V}$_*E*_(ml/min) peak, SpO_2_ (%), and lactate/Tlim (mmol/s) when compared with the SHAM ventilation (Table 1).

**Table 1:** Effects of non-invasive positive pressure ventilation (NIPPV) and SHAM ventilation on selected metabolic, ventilatory, and cardiovascular/hemodynamics at exercise cessation [peak of constant load exercise (CLE)] (*N* = 14).

**Variables**	***NIPPV***	***SHAM***	***p***
IPAP, cmH_2_O	8.6 ± 0.7	4.8 ± 0.5	<0.001
EPAP, cmH_2_O	4.5 ± 0.8	3.0 ± 0	<0.001
Tlim, s	129 ± 29	98 ± 29	0.015
WR, W	44 ± 17	-	-
**Metabolic and ventilatory**
$\overset{\cdot }{V}{\text{O}}_{2}$, mL/min	1,278 ± 581	903 ± 573	0.016
$\overset{\cdot }{V}{\text{O}}_{2}$ (mlO_2_·kg^−1^·min^−1^)	18 ± 8	12 ± 7	0.008
*V°C*O_2_, mL/min	1,105 ± 483	773 ± 433	0.015
*V°_*E*_*, mL/min	33 ± 11	24 ± 12	0.009
**Cardiovascular/hemodynamics**
Heart rate, bpm			
Δ peak—rest, bpm	37 ± 6	34 ± 5	0.588
Δ peak −1′ recovery, bpm	19 ± 4	19 ± 5	0.937
SpO_2_ peak, %	94.7 ± 3.5	92.7 ± 5.2	0.038
SBP peak, mmHg	158 ± 27	155 ± 16	0.512
DBP peak, mmHg	86 ± 15	83 ± 15	0.413
**Blood lactate**
Peak, mmol/l	3.3 ± 1.0	3.2 ± 1.0	0.734
Lactate/time, mmol/s	43 ± 17	33 ± 16	0.028
Borg dyspnea peak	3 (1)	4 (1)	0.305
Borg fatigue peak	3 (1)	4 (2)	0.335

*NIPPV, non-invasive positive pressure ventilation; COPD, chronic obstructive pulmonary disease; HF, heart failure; SpO_2_, peripheral oxygen saturation; WR, work rate; V°O_2_, oxygen uptake; V°CO_2_, carbon dioxide production; V°_E_, ventilation; HR, heart rate; Student's t-test*.

**Figure 3:**
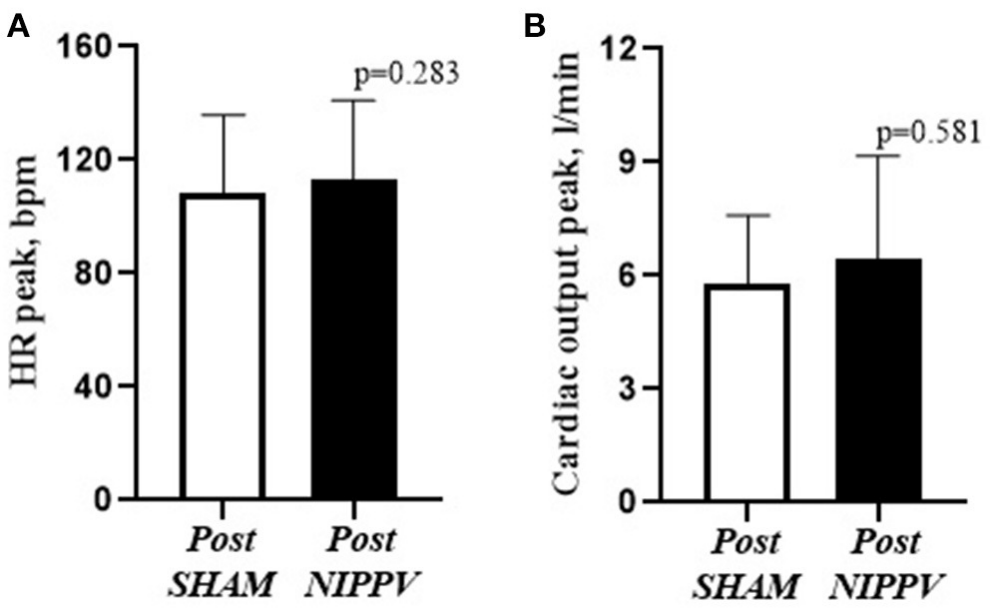
Comparison between HR and cardiac output during ventilation and NIPPV. Heart rate (HR); non-invasive positive pressure ventilation (NIPPV); **(A)** HR peak. **(B)** cardiac output. Student's *t*-test.

There were no significant effects of NIPPV on cardiovascular responses compared with Sham. As shown in Figure 3, cardiac output and heart rate did not differ between interventions at Tlim.

### Effects of NIPPV and SHAM as an Adjunct of High-Intensity Exercise on Cox, Respiratory, and Peripheral Muscle Oxygenation

**Figure 4:**
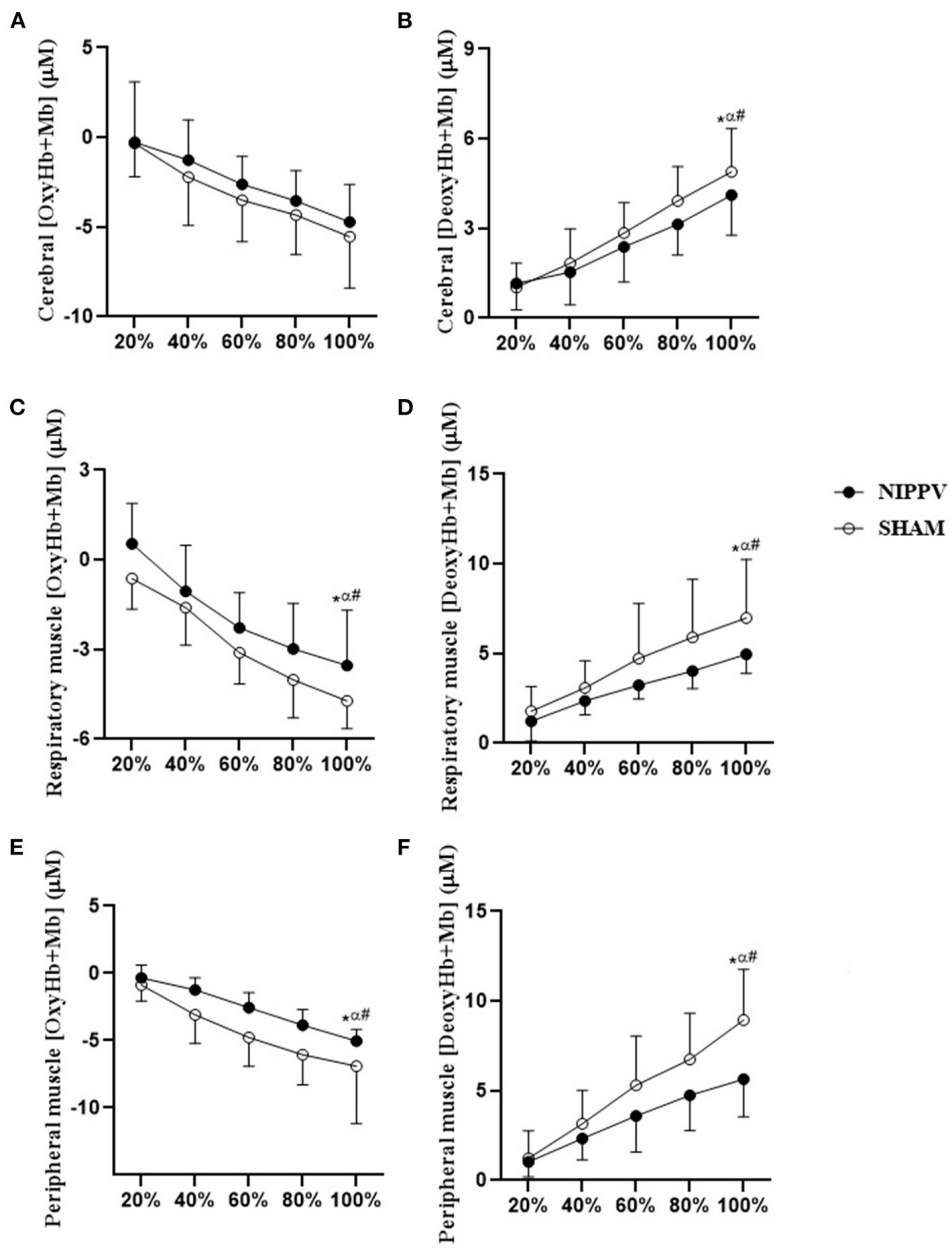
Comparative analysis of the effects of NIPPV (closed symbols) and SHAM ventilation (open symbols) on Cox, respiratory, and peripheral muscle oxygenation. During high-intensity exercise (*N* = 14). **(A)** (OxyHb+Mb) cerebral *p interaction*: 0.87; **(B)** (OxyHb+Mb) peripheral muscle (vastus lateralis muscle) *p interaction*: 0.10; **(C)** (OxyHb+Mb) respiratory muscle (intercostal muscle) *p interaction*: 0.79; **(D)** (DeoxyHb+Mb) cerebral *p interaction*: 0.03; **(E)** (DeoxyHb+Mb) peripheral muscle (right vastus lateralis muscle) *p interaction*: 0.79; **(F)** (DeoxyHb+Mb) respiratory muscle (intercostal muscle) *p interaction* < 0.01. *p < 0.05 100% *NIPPV* vs. 20–40% *NIPPV;* ^α^100% SHAM vs. 60–80% *NIPPV*; ^#^100% *NIPPV* vs. 100% SHAM. Two-way variance analysis with Bonferroni *post-hoc*.

In Cox, respiratory, and peripheral muscles, NIPPV provided an increment in OxyHb+Mb (*p* < 0.05) and an improved deoxygenation response DeoxyHb+Mb (*p* < 0.05) from the half of the test (60%) when compared with the SHAM ventilation; these results corroborate the increase in Tlim with NIPPV (Figure 4).

**Figure 5:**
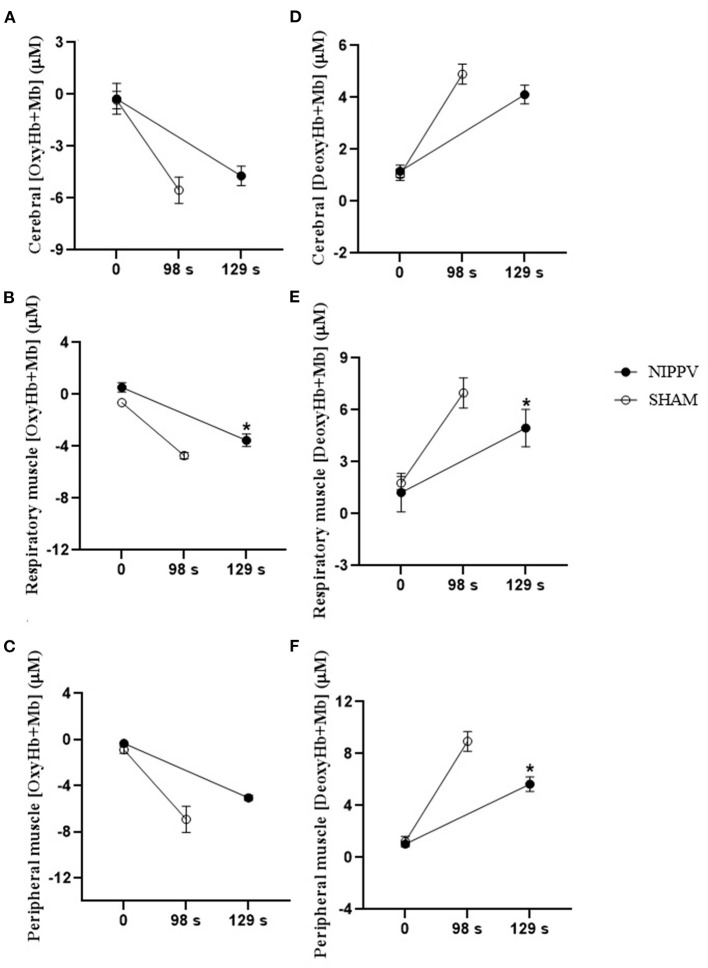
A comparative analysis in Cox, peripheral, and respiratory muscle during rest and limit of tolerance (Tlim) (SHAM 98 s and NIPPV 129 s). *p < 0.05, ANOVA two-way. **(A)** (OxyHb+Mb) cerebral; **(B)** (OxyHb+Mb) peripheral muscle (vastus lateralis muscle); **(C)** (OxyHb+Mb) respiratory muscle (intercostal muscle); **(D)** (DeoxyHb+Mb) cerebral *p* interaction: 0.03; **(E)** (DeoxyHb+Mb) respiratory muscle (intercostal muscle); **(F)** (DeoxyHb+Mb) peripheral muscle (right vastus lateralis muscle).

Figure 5 illustrates the response of DeoxyHb+Mb and OxyHb+Mb at rest (time 0) and Tlim (SHAM 98 s and NIPPV 129 s) in Cox, respiratory, and peripheral muscles. The declines in respiratory and peripheral muscles OxyHb+Mb were greater with NIPPV at a higher Tlim when compared with SHAM (Figures 5B,C). NIPPV provided reduced deoxygenation of respiratory and peripheral muscles at peak exercise when compared with SHAM (Figures 5E,F).

## Discussion

Several aspects make this study are novel and meaningly add to the literature. In particular, this is the first study to investigate the effects of respiratory muscle unloading on respiratory, peripheral muscle, and cerebral oxygenation in patients diagnosed with COPD and HF, a frequently observed multimorbidity phenotype in clinical practice.

The main results are summarized as follows: (1) improvement of exercise tolerance with bilevel NiPPV was observed by a significant improvement in $\overset{\cdot }{V}{\text{O}}_{2}$, SpO_2_, ${\overset{\cdot }{V}}_{\text{E}}$, and blood lactate; (2) respiratory muscle unloading during exercise with NIPPV resulted in an improvement of cerebral, ventilatory, and leg muscle oxygenation in patients with COPD-HF. These data indicated that respiratory muscle unloading through NIPPV resulted in a redirection of blood flow from respiratory to locomotor muscles with positive effects on energy supply to the latter muscle group during exercise.

### Effect of NIPPV on Metabolic and Ventilatory Variables

In our study, we found that NIPPV provided an increase of 6 mlO_2_·kg^−1^·min^−1^ in $\overset{\cdot }{V}{\text{O}}_{2}$, 9 ml/min in $\overset{\cdot }{V}$_*E*_, and 2% in SpO_2_ when compared with SHAM ventilation during exercise. Currently, a minimum detectable difference of 1.02 mlO_2_·kg^−1^·min^−1^ for $\overset{\cdot }{V}{\text{O}}_{2}$ indicates a successful intervention (21), a threshold well-surpassed in the current study supporting the use of NIPPV during exercise in patients with COPD-HF. We believe that the results of the current study can be explained by the following: (1) an increase in pleural pressure fluctuations as a result of increased ventilatory demand during exercise; and (2) the increase of 8–12 cmH_2_O in IPAP and 4–6 cmH_2_O in PEEP increased the cardiac function and improved blood supply, both favorably impacting the exercise performance (21).

The increase in $\overset{\cdot }{V}$_*E*_ and SpO_2_ during exercise with NIPPV may be directly related to the improved ventilation and pulmonary hemodynamics, an improved ventilation-perfusion ratio, and improved control of the deleterious effects of dynamic hyperinflation (22–24). Consistent with these results, we have previously demonstrated that NIPPV modulates the flow mediated vasodilation and improves the blood flow immediately after exercise (8).

### Effect of NIPPV on Respiratory Muscle Unload During Exercise

In our study, we found that NIPPV provided respiratory muscle unloading, contributing to a redistribution of oxygen supply from the respiratory to peripheral musculature (Figure 4). NIPPV was associated with a blunted decrease of respiratory and peripheral muscles OxyHb+Mb during exercise with a consequent increase of DeoxyHb+Mb. Moreover, total body $\overset{\cdot }{V}{\text{O}}_{2}$ was higher after respiratory muscle unloading (Table 1). Taken together, these results indicate that peripheral muscle oxygen consumption is limited by supply when no ventilatory support is administered in patients with COPD-HF. Borghi-Silva et al. (4) first demonstrated that respiratory unloading, as observed when applying proportional assisted ventilation, counterbalanced the potential reductions in muscular $\overset{\cdot }{V}{\text{O}}_{2}$ with positive consequences on respiratory $\overset{\cdot }{V}{\text{O}}_{2}$. We believe that this mechanism is due to blood flow redistribution from the diaphragm to the peripheral muscles, with positive consequences on leg fatigue reduction and longer exercise time when compared with the SHAM intervention (Table 1; Figures 4, 5).

The improvements in tissues oxygenation (i.e., less oxygen extraction, Table 2) following NIPPV, can be partially attributed to improvements in cerebral, respiratory, and cerebral oxygen delivery secondary to greater arterial oxygenation (i.e., SpO_2_). Some studies have already demonstrated a redistribution of blood flow from the peripheral to respiratory muscles during high-intensity exercise, termed “flow steal,” in patients with either COPD or HF (7, 10, 25). This phenomenon is linked to several mechanisms, including ventilatory limitations that contribute to a respiratory muscle fatigue-induced metaboreflex. This mechanism is activated by an increase in sympathetic vasoconstrictor outflow, reducing the perfusion of appendicular muscles during high-intensity exercise.

**Table 2:** Cerebral, respiratory, and peripheral ΔOxyHb+Mb and ΔDeoxyHb+Mb values during high-intensity exercise in patients with COPD-HF.

	**NIPPV**		**SHAM**	
**Time exercise**	**ΔOxyHb+Mb (μM)**	**ΔDeoxyHb+Mb (μM)**	**ΔOxyHb+Mb (μM)**	**ΔDeoxyHb+Mb (μM)**
**Cerebral**
20%	−0.26 ± 0.89	1.15 ± 0.23	−0.33 ± 0.49	1.02 ± 0.21
40%	−1.27 ± 0.60	1.53 ± 0.29	−2.22 ± 0.71	1.83 ± 0.30
60%	−2.61 ± 0.41	2.38 ± 0.31	−3.50 ± 0.61	2.84 ± 0.27^α^
80%	−3.55 ± 0.45	3.13 ± 0.27	−4.33 ± 0.58	3.91 ± 0.30^α^
100%	−4.41 ± 0.55	4.11 ± 0.35	−5.53 ± 0.76	4.89 ± 0.38^#^
**Respiratory muscle**
20%	0.53 ± 0.36	1.22 ± 0.29	−0.62 ± 0.27	1.77 ± 0.36
40%	−1.05 ± 0.41	2.36 ± 0.20	−1.60 ± 0.33	3.08 ± 0.40
60%	−2.27 ± 0.31	3.22 ± 0.20	−3.10 ± 0.28^α^	4.72 ± 0.82^α^
80%	−2.97 ± 0.40	4.01 ± 0.25	−4.01 ± 0.34^α^	5.90 ± 0.86^α^
100%	−3.53 ± 0.49	4.95 ± 0.28	−4.71 ± 0.24^#^	6.98 ± 0.87^#^
**Peripheral muscle**
20%	−0.33 ± 0.25	1.03 ± 0.22	−0.87 ± 0.32	1.22 ± 0.41
40%	−1.23 ± 0.23	2.32 ± 0.31	−3.08 ± 0.57	3.15 ± 0.49
60%	−2.55 ± 0.29	3.59 ± 0.53	−4.76 ± 0.57^α^	5.30 ± 0.73^α^
80%	−3.86 ± 0.31	4.74 ± 0.52	−6.05 ± 0.59^α^	6.73 ± 0.69^α^
100%	−5.04 ± 0.34	5.64 ± 0.56	−6.90 ± 1.13^#^	8.94 ± 0.75^#^

α *100% SHAM vs. 60–80% NIPPV*.

# *100% NIPPV vs. 100% SHAM*.

*Two-way variance analysis with Bonferroni post-hoc. The deltas of the variables were analyzed by near-infrared spectroscopy (NIRS) at the cerebral, peripheral, and respiratory muscles were calculated during CLE, in seconds, divided into deltas [20, 40, 60, 80, and 100% of limit of tolerance (Tlim)], subtracted from the value obtained at rest (60 s of the most stable data)*.

Other potential mechanisms of exercise intolerance in patients with the cardiopulmonary disease have been explored, including the negative impact of cardiovascular hemodynamics, a deficit in oxygen supply with negative consequences on redistribution of O_2_ to muscle during exercise without any device (non-invasive ventilation, oxygen supplementation, or heliox) to facilitate respiratory muscle unloading (7, 10, 25). Accordingly, we reinforce the importance of future clinical trials of the impact of this kind of intervention considering the different patient phenotypes to better understand the mechanisms of improvement of exercise tolerance and better understand the effectiveness of different non-invasive ventilation approaches for clinical application.

We found an improvement in Cox with NIPPV, patients had greater O_2_ extraction when applied to SHAM ventilation, demonstrating that NIPPV also provided a “restoration” in Cox. Patients with COPD-HF have a reduction in the resting Cox and may be worsened during exercise. These abnormalities may prevent COx from increasing during exercise, a likely consequence of the combined deleterious effects of systemic hemodynamic compromise (9).

### Limitation of the Study

This study has some limitations which are inherent to the patient cohort assessed. All patients had an overlap disease diagnostic (i.e., COPD and HF), however, they were not similar in terms of severity of the respiratory and cardiovascular disease. Patients requiring oxygen therapy and presenting with other associated comorbidities, which would both indicate higher disease severity, were excluded. Therefore, interpretation of our findings may be limited to patients with COPD-HF with mild disease severity. Furthermore, it is important to apply the same study protocol in patients with HF and COPD isolated. Another important limitation to be considered is the lack of application of indocyanine green dye to assess cerebral oxygenation. Other studies have evaluated cerebral oxygenation during exercise using this invasive method, however, these investigations have been applied with only few patients (26, 27). Considering the non-invasive nature of the present study, our findings of cerebral oxygenation analysis require further investigation in the future. Another limitation of our study was not evaluating the variable absolute index of local tissue oxygen saturation (StiO_2_ %) due to technical problems in our device.

Other limitations of our study were the absence of comparisons at an isotime point of exercise with and without NIPPV to get a better insight of the contribution of each system, i.e., cardiovascular, respiratory, and local tissues (respiratory muscle, locomotor muscles, and cerebral cortex); and the absence of variable absolute index of local tissue oxygen saturation (StiO_2_ %) which is commonly adopted as an index of tissue oxygen availability, reflecting the balance between the muscle oxygen supply and demand (28).

### Clinical Implications

Our results confirm that NIPPV can help to sustain higher levels of exercise training intensity, which may allow a short-term exercise training program to have enhanced positive effects on the peripheral and respiratory muscle oxygenation, metabolic and ventilatory responses, and greater exercise time during exercise.

Therefore, the effects of NIPPV provided greater oxygen utilization in peripheral musculature during exercise, directly contributing to greater exercise tolerability in patients with COPD-HF.

## Conclusion

Non-invasive positive pressure ventilation as an adjunct of high-intensity exercise unloads the respiratory musculature, enhancing oxygen supply to the peripheral musculature, providing and reducing the muscle fatigue longer exercise time in patients diagnosed with COPD and HF.

## Data Availability Statement

The raw data supporting the conclusions of this article will be made available by the authors, without undue reservation.

## Ethics Statement

The studies involving human participants were reviewed and approved by the Local Ethics Committees (Protocol Number: 91088318.7.1001.5504) of University of São Carlos. The patients/participants provided their written informed consent to participate in this study.

## Author Contributions

All authors approved the final manuscript and contributed to the conception of the work, the acquisition and analysis of data, and drafting and revising the intellectual content.

## Funding

This study was supported by a research grant from the Fundação de Amparo à Pesquisa do Estado de São Paulo, São Paulo, Brazil (FAPESP) Process No. 2015/26/501-1 and by the Coordenação de Aperfeiçoamento de Pessoal de Nível Superior- Brasil (CAPES - 001) and CNPq: 141803/2019-3. CG was a doctorate student supported by the FAPESP, Process Nos. 2018/03233-0 and 2020/13465-5. AB-S was an established Investigator (level IB) of the Conselho Nacional de Desenvolvimento Científico e Tecnológico (CNPq), Brazil.

## Conflict of Interest

The authors declare that the research was conducted in the absence of any commercial or financial relationships that could be construed as a potential conflict of interest.

## Publisher's Note

All claims expressed in this article are solely those of the authors and do not necessarily represent those of their affiliated organizations, or those of the publisher, the editors and the reviewers. Any product that may be evaluated in this article, or claim that may be made by its manufacturer, is not guaranteed or endorsed by the publisher.
